# Sulphate of Spartein in Surgical Practice

**Published:** 1907-03

**Authors:** Stuart McGuire

**Affiliations:** Surgeon in Charge St. Luke’s Hospital Richmond, Va.


### SULPHATE OF SPARTEIN IN SURGICAL PRACTICE.*

By Stuart McGuire, M. D., Surgeon in Charge St. Luke's Hospital, Richmond, Va.

Like most surgeons, I devote little time to the study of the therapeutic action of drugs. Patients who are referred to me have usually exhausted the resources of materia medica, and in my practice I rarely have occasion to employ medicinal agents other than the well known anæsthetics, antiseptncs, purgatives and tonics. I believe, however, I have accidentally discovered in Sulphate of Spartein a valuable remedy for the prevention and treatment of post-operative suppression of urine.

I do not know whether my experience coincides with that of other surgeons, but it is a fact that in the last five years I have lost more cases from post-operative suppression of urine than from all other causes combined, and this despite the almost routine use of chloroform as an anæsthetic.

The cases have usually been those with pre-existing nephritis from sepsis of cholemia. Shock has not apparently been a factor, as the condition would not develop for twenty-four or thirty-six hours. A patient operated on for retention of urine, or for jaundice due to obstruction of the common duct, would do well for one or two days and just as he was thought to be out of danger there would come the news that he was passing no urine. He would become restless, then

*Read before the Southern Surgical and Gynecological Association, held in Baltimore, Md., December 11, 12, 13, 1906.

listless, would develop a stupor which would rapidly deepen into coma, and would die with all the symptoms characteristic of uremia. In the treatment of this condition I tried water by mouth, under the skin and in the rectum; hot packs and vapor baths; cups and counter irritants; strychnia, digitalis and nitro-glycerine; calomel and saline purgatives; and in one case stripping the kidney capsules, with uniformly bad results.

Two years ago I began empirically the use of sulphate of spartein and I now have the record of six cases in which I am sure the drug was the means of saving the patient's life.

I will not occupy the time of the Association by reading a dissertation on spartein which I would, of course, have to copy from a text-book. Its therapeutic effect is to increase the blood pressure, make the pulse slower and stronger, and act as a powerful diuretic. Its action is manifest in 30 minutes after administration and lasts for four or six hours.

I believe the reason why the value of sulphate of spartein is not more widely recognized is because authorities advise its use in doses so small as to be worthless. To get results it must be given hypodermically in from one to two grains, repeated every three to six hours. When so employed I have repeatedly seen it pull up a runaway heart and set in motion a pair of stalled kidneys. Its use should not be delayed until suppression of urine is already in existence, but it should be prescribed as a prophylactic as well as a curative agent.

I do not mean to claim it is a specific, or that it should be employed to the exclusion of other measures, such as purgatives, transfusions, and hot packs. I do believe, however, from actual experience that it is preferable to the drugs of the digitalis type in rapidity of action, ease of administration, and what is more important—efficiency of results.—Va. Med. Semi-Monthly.

